# Calcium Ion-Induced Self-Assembly of Carboxylated Polyallylamine-*graft*-Poly(Ethylene Glycol) in an Aqueous Medium

**DOI:** 10.3390/polym17233129

**Published:** 2025-11-25

**Authors:** Junya Emoto, Yukiya Kitayama, Atsushi Harada

**Affiliations:** Department of Applied Chemistry, Graduate School of Engineering, Osaka Metropolitan University, 1-1 Gakuen-cho, Naka-ku, Sakai 599-8531, Japan; su23352k@st.omu.ac.jp (J.E.); kitayama@omu.ac.jp (Y.K.)

**Keywords:** double hydrophilic copolymer, self-assembly, calcium ion, graft copolymer

## Abstract

Double hydrophilic copolymers (DHCs) can form nano-assemblies such as micelles and vesicles in aqueous media under certain environmental conditions. These assemblies have attracted much attention in both fundamental and applied research. To date, most studies on DHC self-assemblies have focused on block copolymers rather than graft copolymers. In this study, we investigated using Ca^2+^ ions in an aqueous medium to induce the formation of carboxylated polyallylamine-*graft*-poly(ethylene glycol) (PAA-g-PEG) self-assemblies as a graft-type DHC. Dynamic light scattering measurements conducted under various conditions showed that the carboxylated PAA-g-PEG self-assemblies had a micellar structure with a core of Ca^2+^ ions/carboxylates surrounded by non-ionic poly(ethylene glycol) grafts. Confocal laser scanning microscopy showed that the carboxylated PAA-g-PEG self-assemblies were able to deliver Ca^2+^ ions into cells. These results show that carboxylated PAA-g-PEG self-assemblies formed in the presence of divalent metal ions have potential for future applications in the biomedical field.

## 1. Introduction

Double hydrophilic copolymers (DHCs) have attracted much attention [1,2,3,4,5,6,7,8,9,10,11,12,13,14,15,16,17,18,19,20,21] because they are normally hydrophilic and can form nano-assemblies such as micelles and vesicles in aqueous media with changes in the pH [3,4,9,10,11,13,14,15,16], temperature [1,5,6,9,10,11,13,14,15,16,17], ionic strength [3,7], physicochemical interactions [12,18,19,20,21], and/or miscibility of polymers [8,17]. To date, most research on DHC self-assemblies has focused on block copolymers, and reports on graft copolymers are limited. As an example of DHC self-assembly using a graft copolymer in an aqueous medium, Glaive et al. reported on the self-assembly behavior of poly(2-methyl-2-oxazoline-co-2-pentyl-2-oxazoline)-*graft*-poly(2-ethyl-2-oxazoline) using the immiscibility between poly(2-methyl-2-oxazoline-co-2-pentyl-2-oxazoline) and poly(2-ethyl-2-oxazoline) [17]. The poly(2-methyl-2-oxazoline-co-2-pentyl-2-oxazoline)-*graft*-poly(2-ethyl-2-oxazoline) self-assemblies were amphiphilic copolymers at temperatures above the lower critical solution temperature because of the thermoresponsive property of poly(2-ethyl-2-oxazoline), and this led to micellization via hydrophobic interactions. Zhao et al. reported pH/thermo-double responsive self-assembly behavior using poly(L-glutamic acid)-*graft*-poly(*N*-isopropylacrylamide) (PGA-g-PNIPAM) [16]. PGA-g-PNIPAM self-assembled above the lower critical solution temperature of poly(*N*-isopropylacrylamide) grafts at pH 5.5 and 7.4. Under acidic conditions (pH 5.25 and 4.6), PGA-g-PNIPAM was soluble because of conformational changes in the PGA chains.

One strategy for self-assembly of DHCs is to induce cross-linking between two anionic carboxylates using a cationic divalent metal ion, which leads to gelation and association of multivalent carboxylates, as in alginate gels [22]. As an example of this, Li et al. developed a self-assembling system of poly(ethylene oxide)-*block*-polymethacrylate copolymer using Ca^2+^ or Ba^2+^ ions [18]. The poly(ethylene oxide)-*block*-polymethacrylate copolymer was soluble in pure water or monovalent alkaline metal ion solutions. Addition of alkaline earth metal ions (e.g., Ca^2+^ or Ba^2+^) made the PMA block insoluble because of neutralization, and this resulted in the formation of nano-aggregates that were sterically stabilized by soluble PEO blocks. To date, no studies have reported on the self-assembly of graft-type DHCs induced by metal ions in aqueous media. Furthermore, the polymers forming complexes with metal ions, including block- and graft-type DHC self-assembly, are expected to find applications in environmental purification and medical fields, such as the selective adsorption and removal of metal ions, as well as metal ion delivery.

**Figure 1:**
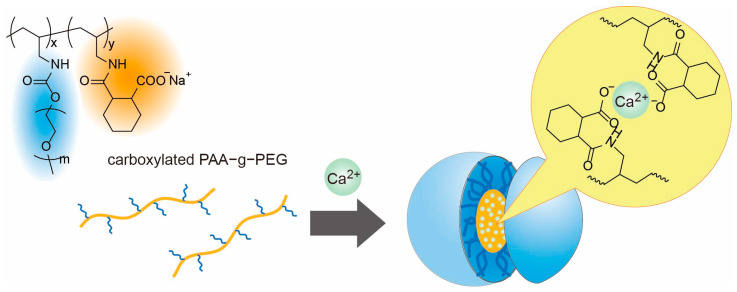
Self-assembly of carboxylated PAA-g-PEG induced by the interaction between carboxylates and Ca^2+^ ions.

Herein, we investigated the Ca^2+^ ions-induced self-assembly of the graft-type DHC carboxylated polyallylamine-*graft*-poly(ethylene glycol) (carboxylated PAA-g-PEG) (Figure 1). Carboxylated PAA-g-PEG contains hydrophilic and non-ionic poly(ethylene glycol) (PEG) grafts and carboxylates introduced in PAA side chains via a cyclohexyl spacer. The effects of ion valence and concentration on the self-assembly of carboxylated PAA-g-PEG and the effect of pH on the size of carboxylated PAA-g-PEG self-assembly with a micellar structure were investigated using mainly light-scattering techniques. Finally, the Ca^2+^ ion delivery of carboxylated PAA-g-PEG self-assembly into the cultured cells was evaluated using confocal laser scanning microscopic observation.

## 2. Materials and Methods

### 2.1. Materials

Polyallylamine (MW = 100,000) was kindly provided by Nittobo (Tokyo, Japan). NaCl, Na_2_CO_3_, NaOH, LiCl, HCl (35% mass fraction), methanol, Dulbecco’s modified Eagle medium, RPMI 1640, and Dulbecco’s phosphate-buffered saline (DPBS) were purchased from Nacalai Tesque, Inc. (Kyoto, Japan). CaCl_2_, NaHCO_3_, anhydrous tetrahydrofuran (THF), ethyl acetate, acetone, and pyrene were purchased from FUJIFILM Wako Pure Chemical Corp. (Osaka, Japan). *cis*-Cyclohexane-1,2-dicarboxylic anhydride (CHex), α-methyl-ω-hydroxy poly(ethylene glycol) (*M*_n_ = 550), 4-nitrophenyl chloroformate, and triethylamine were purchased from Sigma–Aldrich (St. Louis, MO, USA). Fluo 3-AM was purchased from DOJINDO Laboratories Co., Ltd. (Kumamoto, Japan).

### 2.2. Synthesis of Carboxylated PAA-g-PEG

PAA-g-PEG was synthesized according to our previous report [23], and carboxylated PAA-g-PEG was then synthesized by carboxylation of primary amines into the side chains of PAA.

#### 2.2.1. Synthesis of α-methyl-ω-nitrophenyl Carbonate PEG

First, a solution of α-methyl-ω-hydroxy PEG (5.112 g, 9.29 mmol) and triethylamine (5.8 mL, 41.8 mmol) was prepared in anhydrous THF (51.1 mL). Next, 4-nitrophenyl chloroformate (5.62 g, 27.9 mmol) was dissolved in anhydrous THF (74.4 mL) and then added dropwise to the PEG solution over 1 h. The mixture was stirred at room temperature for 3 days, and a precipitate formed, which was removed by filtration. The filtrate was concentrated under reduced pressure to give a crude, light-yellow, viscous liquid. This liquid was redissolved in a small volume of ethyl acetate and purified using silica-gel flash column chromatography with ethyl acetate and then methanol. The fraction was dried under reduced pressure to obtain α-methyl-ω-nitrophenyl carbonate PEG as a light-yellow, viscous liquid (yield: 4.98 g, 71%). Successful synthesis and purification were confirmed by ^1^H NMR (JNM-LA300 FT-NMR system, JEOL Ltd., Tokyo, Japan).

#### 2.2.2. Synthesis of PAA-g-PEG

First, a solution of PAA (793 mg) was prepared in a 25 mM LiCl solution in methanol (793 mL). Next, α-methyl-ω-nitrophenyl carbonate PEG (1242 mg) was dissolved in 25 mM LiCl in methanol (88 mL), and the resulting solution was added dropwise to the PAA solution. The mixture was stirred at room temperature for 3 days and then dialyzed (Spectra/Por Membrane, molecular weight cutoff [MWCO]: 12–16 kDa, Spectrum Chemical, New Brunswick, NJ, USA) against dilute aqueous HCl (pH 4–5) for 2 days. This was followed by lyophilization to obtain a yellow solid. The solid was redissolved in a small volume of dilute aqueous HCl (pH 4–5) and purified by cation-exchange column chromatography (Macro-Prep High S Media; Bio-Rad Laboratories, Inc., Hercules, CA, USA) using dilute HCl (pH 4–5) followed by NaCl aq. (2 M, pH 11). The obtained fraction was neutralized with aqueous HCl (1 M) and concentrated under reduced pressure. The resulting solution was dialyzed against dilute HCl (pH 4–5) for 2 days and then lyophilized to obtain PAA-g-PEG as a white solid (yield: 1113 mg, 74%). The successful synthesis and purification were confirmed by ^1^H NMR.

#### 2.2.3. Carboxylation of Primary Amines in PAA-g-PEG

PAA-g-PEG was dissolved in carbonate buffer containing 500 mM NaCl (100 mM, pH 9.5, 76.3 mL), and the pH was adjusted to 9.5 using 1.0 M NaOH aq. Five equivalents of CHex relative to the primary amines were added to the PAA-g-PEG solution over three steps (one equivalent, two equivalents, two equivalents), with each addition separated by 1 h. Before each addition of Chex, the pH was adjusted to 9.5 using 1.0 M NaOH aq. After stirring for 24 h at room temperature, 100 equivalents of NaCl relative to the primary amines were added to the solution. The solution was then stirred overnight at room temperature, dialyzed (Spectra/Por Membrane, molecular weight cutoff [MWCO]: 12–16 kDa, Spectrum Chemical) against dilute aqueous NaOH (pH 9–10) for 3 days, and lyophilized. Carboxylated PAA-g-PEG was obtained as a white solid (yield: 714 mg, 98%).

### 2.3. Characterization of the Carboxylated PAA-g-PEG Self-Assembly

#### 2.3.1. Effect of Salt on Self-Assembly of Carboxylated PAA-g-PEG

Carboxylated PAA-g-PEG (5.0 mg/mL) was dissolved in NaCl or CaCl_2_ aq. (10, 20, 30, 60, 90, 120, or 150 mM). The pH of each solution was adjusted to 7.4. Dynamic light scattering (DLS) measurements of the prepared solutions were performed using a Zetasizer Nano ZS (Spectris Co., Ltd., Kanagawa, Japan) at 25 °C. Solutions were also prepared containing ethylenediaminetetraacetic acid (EDTA). First, carboxylated PAA-g-PEG (5.0 mg/mL) was dissolved in a 150 mM CaCl_2_ aq. and the pH was then adjusted to 7.4. Next, aqueous EDTA (equimolar to CaCl_2_) was added to the carboxylated PAA-g-PEG aqueous solution. DLS measurements were performed after storing the mixture overnight.

#### 2.3.2. Effect of the Polymer Concentration on Self-Assembly of Carboxylated PAA-g-PEG

To prepare solutions with various polymer concentrations (1.0, 2.0, 3.0, 4.0, or 5.0 mg/mL), carboxylated PAA-g-PEG was dissolved in a 150 mM CaCl_2_ aq. and the pH was adjusted to 7.4. DLS measurements of the prepared solutions were performed at 25 °C.

#### 2.3.3. Determination of the Critical Micelle Concentration of the Carboxylated PAA-g-PEG Self-Assemblies

An acetone solution of pyrene (0.6 mM, 3 μL) was pipetted into a series of glass vials, followed by the evaporation of acetone. The polymer solutions (3.0 mL) with varying concentrations were then added to each of the vials. The pyrene concentration in the final solution was adjusted to the saturated solubility of pyrene in water at 25 °C. After incubation of the vials for 48 h in the dark, fluorescence spectra (*λ*_ex_ = 337 nm) were measured using a spectrofluorometer (FP-8500, JASCO, Tokyo, Japan) at 25 °C.

#### 2.3.4. Acid–Base Titration of Carboxylated PAA-g-PEG

Carboxylated PAA-g-PEG (80 mg) was dissolved in a 150 mM CaCl_2_ aq. (12 mL). The pH was then adjusted to 11 using 1 M NaOH aq. This polymer solution was titrated with 150 mM HCl containing 150 mM CaCl_2_ using an automatic titrator (AUT-701, DKK-TOA Corp., Tokyo, Japan). The CaCl_2_ concentration in the polymer solution was maintained at 150 mM during the titration.

#### 2.3.5. Intracellular Delivery of Ca^2+^ Ions by the Carboxylated PAA-g-PEG Self-Assembly

Carboxylated PAA-g-PEG (5.0 mg/mL) was dissolved in a 150 mM CaCl_2_ aq. and the pH was then adjusted to 7.4. To remove free Ca^2+^ ions and exchange the solvent to DPBS, the polymer solution was processed three times by ultrafiltration (Amicon Ultra, MWCO: 3000 Da, Sigma–Aldrich). DLS measurements were then performed on the polymer solution, and the Ca^2+^ ion concentration was further determined by inductively coupled plasma atomic emission spectroscopy (SPS7800, Seiko Instruments Inc., Chiba, Japan). HeLa cells (human cervical cancer cells) were seeded in a glass-bottom dish at 2.0 × 10^5^ cells per dish and incubated for 24 h at 37 °C. The cells were washed twice with DPBS, followed by the addition of fresh medium containing carboxylated PAA-g-PEG self-assemblies (1.0 mg/mL) or a DPBS solution of CaCl_2_ (372 µM). After incubation for 4 h, the cells were washed twice with DPBS, followed by the addition of fresh medium containing Fluo 3-AM (4.0 µM) and another 90 min of incubation at 37 °C. After washing three times, the treated cells were observed using confocal laser scanning microscopy (CLSM; LSM5 Exciter, Zeiss, Oberkochen, Germany). The cell viability was evaluated by MTT assay, which is based on the conversion of MTT (3-(4,5-dimethyl-2-thiazolyl)-2,5-diphenyltetrazolium bromide) to insoluble formazan crystals by mitochondrial oxidoreductase in living cells. After incubation of the cells with the sample for 4 h, the cells were washed twice with DPBS, followed by the addition of fresh medium containing MTT and another 3 h of incubation at 37 °C. After washing twice, DMSO was added, and the absorbance derived from formazan was observed using a microplate reader (SH-8000Lab, CORONA ELECTRIC Co., Ltd., Ibaraki, Japan).

## 3. Results and Discussion

### 3.1. Synthesis of Carboxylated PAA-g-PEG

**Scheme 1:**
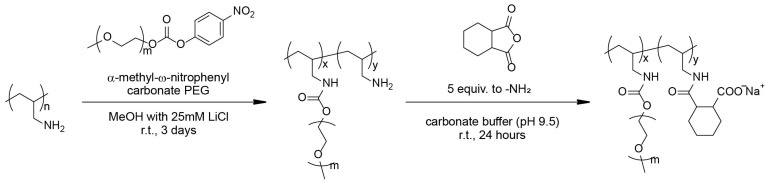
Synthesis of carboxylated PAA-g-PEG.

Carboxylated PAA-g-PEG was synthesized by carboxylation of primary amines in PAA-g-PEG. Briefly, carboxylated PAA-g-PEG was prepared by reaction of primary amine in PAA with α-methyl-ω-nitrophenyl carbonate poly(ethylene glycol) (Figure S1), followed by addition of CHex (Scheme 1). The content of PEG graft was calculated to be 9% to the allylamine unit from the peak area ratio of methylene protons (2.5 ppm and 3.1 ppm) in side chain of PAA (Figure S2), and the completion of conversion of residual primary amines to carboxylates was then confirmed from the peak area ratio of methoxy protons of PEG grafts (4.2 ppm) and the cyclohexyl protons (2.6 ppm) (Figure S3).

### 3.2. Ca^2+^ Ion-Induced Self-Assembly of Carboxylated PAA-g-PEG

**Figure 2:**
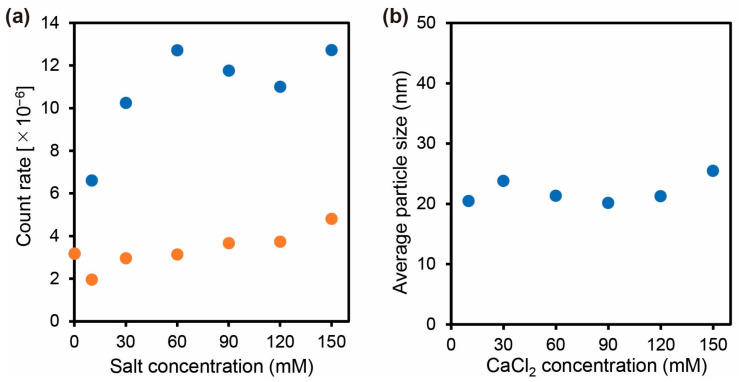
Changes in the count rate (**a**) and average particle size (**b**) with the salt concentration (CaCl_2_, blue circles; NaCl, orange circles) for carboxylated PAA-g-PEG solutions. Conditions: polymer concentration, 5.0 mg/mL; pH, 7.4; and temperature, 25 °C.

The self-assembly of carboxylated PAA-g-PEG in an aqueous medium containing NaCl or CaCl_2_ at pH 7.4 was evaluated by DLS (Figure 2). The count rate stayed almost constant for carboxylated PAA-g-PEG as the NaCl concentration changed, and showed that the count rate was independent of the NaCl concentration for the association of carboxylated PAA-g-PEG. By contrast, the count rate for carboxylated PAA-g-PEG increased with increases in the CaCl_2_ concentration. This difference in the effect obtained with NaCl and CaCl_2_ indicates that the Ca^2+^ ions can induce intermolecular association of carboxylated PAA-g-PEG. At a CaCl_2_ concentration of >60 mM, not only the count rate but also the average particle size was almost constant, which suggested that the association state of carboxylated PAA-g-PEG was similar regardless of the CaCl_2_ concentration. Furthermore, the addition of EDTA, a typical metal ion chelating agent that strongly binds to Ca^2+^ ions, to the carboxylated PAA-g-PEG solution containing 150 mM CaCl_2_ resulted in a decrease in the count rate (Figure S4). This decrease was attributed to the Ca^2+^ ions, which induced the intermolecular association of carboxylated PAA-g-PEG, preferentially interacting with EDTA, which undermined the association between the polymers. This result strongly supports the conclusion that Ca^2+^ ions induced the intermolecular association of carboxylated PAA-g-PEG.

**Figure 3:**
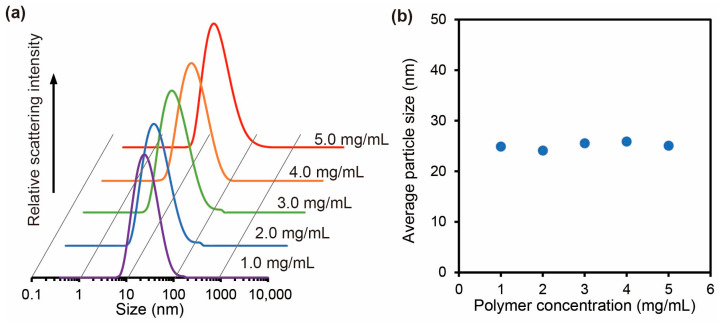
Effect of the polymer concentration on the size distribution (**a**) and average particle size (**b**) of the carboxylated PAA-g-PEG self-assemblies prepared using Ca^2+^ ions. Conditions: CaCl_2_ concentration, 150 mM; pH 7.4; and temperature, 25 °C.

To evaluate the association state of carboxylated PAA-g-PEG with a CaCl_2_ concentration of >60 mM, the effect of the polymer concentration on the average particle size was examined at 150 mM CaCl_2_, which is a sufficient CaCl_2_ concentration (Figure 3). The average particle size remained almost constant regardless of the polymer concentration, which showed that increases in the polymer concentration did not induce further aggregation. This result was attributed to the carboxylated PAA-g-PEG self-assemblies being surrounded by PEG grafts that inhibited further self-assembly [24]. Therefore, the results showed that the carboxylated PAA-g-PEG self-assemblies had a micellar structure.

### 3.3. Characterization of the Carboxylated PAA-g-PEG Self-Assemblies

**Figure 4:**
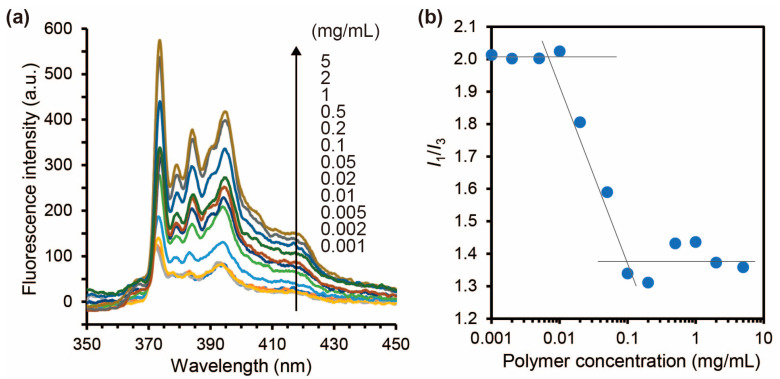
Critical micelle concentration determined using pyrene for the carboxylated PAA-g-PEG self-assemblies formed in the presence of Ca^2+^ ions. Fluorescence spectra of pyrene at various polymer concentrations (**a**). Change in *I*_1_/*I*_3_ values with the polymer concentration (**b**). Conditions: λ_ex_, 337 nm; CaCl_2_ concentration, 150 mM; pH 7.4; and temperature, 25 °C.

The critical micelle concentration (CMC) of the carboxylated PAA-g-PEG self-assemblies was evaluated using pyrene as a fluorescent probe. The *I*_1_ (λ_em_ = 373 nm)/*I*_3_ (λ_em_ = 384 nm) values reflect changes in the microscopic polar environment around the pyrene molecule and were plotted against the polymer concentrations [25,26,27]. A sharp decrease in the *I*_1_/*I*_3_ value with the polymer concentration was observed at approximately 30 μg/mL (Figure 4b), which indicated that the carboxylated PAA-g-PEG self-assemblies in a 150 mM CaCl_2_ aqueous solution exhibited CMC behavior. At a high polymer concentration, the *I*_1_/*I*_3_ values were close to 1.38, which is similar to the value previously measured in ethyl acetate (*I*_1_/*I*_3_ = 1.37) [28] and indicates that the self-assemblies contained a hydrophobic domain. The results (Figure 3 and Figure 4) suggested that the carboxylated PAA-g-PEG self-assemblies formed a micellar structure with a hydrophobic core consisting of carboxylated PAA and Ca^2+^ ions that was surrounded by PEG grafts.

**Figure 5:**
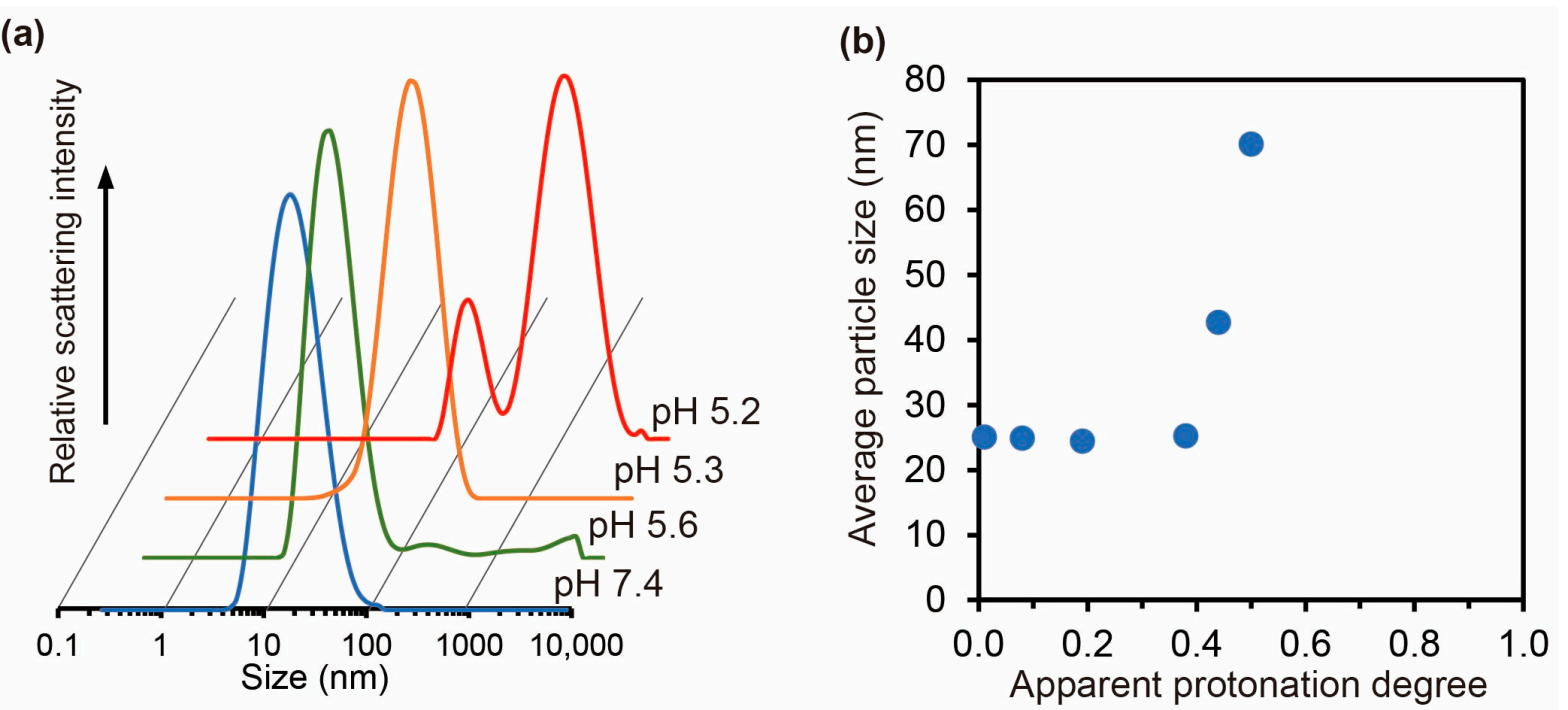
Effect of pH on the size distribution (**a**) and average particle size (**b**) of the carboxylated PAA-g-PEG self-assemblies formed in the presence of Ca^2+^ ions. Conditions: polymer concentration, 5.0 mg/mL; CaCl_2_ concentration, 150 mM; and temperature, 25 °C. In (**b**), the protonation degree was determined by acid–base titration as shown in Figure S4.

The results obtained in Figure 2, Figure 3 and Figure 4 were obtained at pH 7.4, where almost all carboxylates in the side chain of carboxylated PAA-g-PEG will be ionized. It is expected that the interaction between the polymer and Ca^2+^ ions will be affected by the ionization state of the carboxylates and, therefore, the pH. Consequently, we evaluated the effect of pH on the average particle size of the carboxylated PAA-g-PEG self-assemblies using DLS measurements and acid–base titration (Figure 5). There was almost no change in the size distribution at pH 7.4 and 5.6, but clear changes in the size distribution were observed at pH 5.3 and 5.2 compared with that at pH 7.4 (Figure 5a). To study the relationship between the effect of pH and the ionization state of carboxylated PAA-g-PEG, acid–base titration of carboxylated PAA-g-PEG was performed in the presence of 150 mM CaCl_2_ (Figure S5). The average particle size was plotted against the protonation degree (Figure 5b). A steep increase in the average particle size occurred when the protonation degree was approximately 0.5, that is, near the apparent p*K*a of carboxylated PAA-g-PEG. In the pH range above the p*K*a, the number of ionized carboxyl groups will be high, while the number of protonated groups will be low. In this pH range, no change in the average particle size was observed in the present study, which indicates that the carboxylated PAA-g-PEO self-assemblies may have a micellar structure similar to that at pH 7.4. By contrast, when the pH is lower than the p*K*a, more carboxyl groups are protonated than ionized. Here, it is necessary to consider that PEG and polycarboxylates such as poly(acrylic acid) form hydrogen-bonded complexes under low pH conditions [29,30,31]. In the pH range below the p*K*a of carboxylated PAA-g-PEG, the number of carboxylates that can form hydrogen bonds with ether oxygens in PEG is greater than the number of carboxylates that can interact with Ca^2+^ ions. Hydrogen bonding between the protonated carboxylate and ether oxygen in PEG may have a non-negligible effect on the formation of carboxylated PAA-g-PEG self-assemblies. This influence might be observed as an increase in the average particle size with the formation of larger aggregates (Figure 5).

### 3.4. Intracellular Delivery of Ca^2+^ Ions by the Carboxylated PAA-g-PEG Self-Assembly

We evaluated whether the carboxylated PAA-g-PEG self-assemblies could function as a carrier of Ca^2+^ ions using cultured cells (HeLa cells). Samples for the intracellular delivery experiments were processed by ultrafiltration to remove free Ca^2+^ ions, and the resulting solution was solvent exchanged with DPBS. The size distribution of the carboxylated PAA-g-PEG self-assemblies did not change significantly after ultrafiltration, which indicated that the self-assemblies were stable even after the removal of excess Ca^2+^ ions. For a solution without polymer, ultrafiltration reduced the Ca^2+^ ion concentration to below 10 μM. For the carboxylated PAA-g-PEG self-assemblies’ solution, the Ca^2+^ ion concentration measured by inductively coupled plasma atomic emission spectroscopy after ultrafiltration was 1.86 mM. This result is consistent with the formation of carboxylated PAA-g-PEG self-assemblies induced by interaction with Ca^2+^ ions. Furthermore, it was confirmed that carboxylated PAA-g-PEG self-assemblies remained stable with no significant change in average particle size for up to 24 h, even after removing excess Ca^2+^ ions (Figure S7).

**Figure 6:**
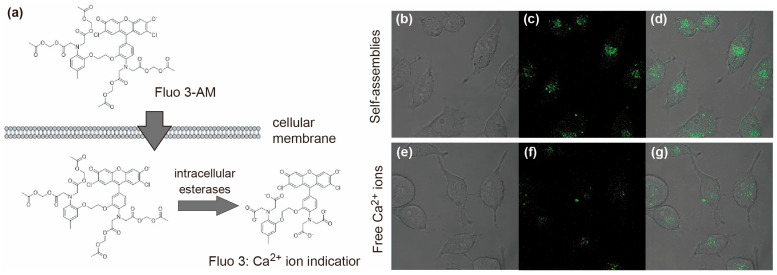
Delivery of Ca^2+^ into HeLa cells by carboxylated PAA-g-PEG self-assemblies. After incubation for 4 h, the cells were treated with Fluo 3-AM. (**a**) Imaging of intracellular Ca^2+^ ions using Fluo 3-AM. (**b**,**e**) Differential interference contrast images. (**c**,**f**) Confocal laser scanning microscopy images. (**d**,**g**) Overlaid differential interference contrast and confocal laser scanning microscopy images.

Intracellular delivery of Ca^2+^ ions by the carboxylated PAA-g-PEG self-assemblies was evaluated by CLSM using Fluo 3-AM, which is the acetoxymethyl ester of the Ca^2+^ ion indicator Fluo 3 (Figure 6a). Fluo 3-AM can penetrate cell membranes because of its high lipid solubility and is converted to Fluo 3 through hydrolysis of acetoxymethyl ester moieties by intracellular esterases. Because Fluo 3 does not easily leak out of cells, it accumulates inside the cells where it binds to intracellular Ca^2+^ ions and emits green fluorescence. CLSM images were obtained of HeLa cells treated with Fluo 3-AM after incubation with the carboxylated PAA-g-PEG self-assemblies for 4 h (Figure 6b–g). Cells treated with carboxylated PAA-g-PEG self-assemblies produced stronger green fluorescence than cells treated with free Ca^2+^ ions. These results demonstrate successful intracellular delivery of Ca^2+^ ions by the carboxylated PAA-g-PEG self-assemblies. Additionally, the cell viability assessed by the MTT assay for the cells treated with carboxylated PAA-g-PEG self-assemblies under the same conditions as Figure 6 was 91.1 ± 3.2% (*n* = 3), and carboxylated PAA-g-PEG self-assemblies had negligible cytotoxicity.

## 4. Conclusions

In this study, we found that the graft-type DHC carboxylated PAA-g-PEG formed self-assemblies through intermolecular association induced by Ca^2+^ ions in an aqueous medium. It is likely that this self-assembly into micellar structures can also be achieved using other multivalent anions that can induce intermolecular association of polymers. Even after removing free Ca^2+^ ions, the carboxylated PAA-g-PEG self-assembly exhibited sufficient stability to deliver Ca^2+^ ions into cells under physiological conditions. An increase in Ca^2+^ ions in the cytoplasm plays a crucial role in cells for functions related to activation of cellular processes [32,33], bone cell proliferation [34,35], and cancer therapy [36,37,38]. The carboxylated PAA-g-PEG self-assemblies prepared in this study using Ca^2+^ show potential for future applications in the biomedical field.

## Data Availability

The original contributions presented in this study are included in the article. Further inquiries can be directed to the corresponding author.

## Supplementary Materials

The following supporting information can be downloaded at https://www.mdpi.com/article/10.3390/polym17233129/s1, Figure S1: ^1^H NMR of α-methyl-ω-nitrophenyl carbonate poly(ethylene glycol); Figure S2: ^1^H NMR of PAA-g-PEG; Figure S3: ^1^H NMR of carboxylated PAA-g-PEG; Figure S4: Count rates of carboxylated PAA-g-PEG self-assemblies in CaCl_2_ aqueous solutions with and without EDTA; Figure S5: Acid–base titration curve of carboxylated PAA-g-PEG in 150 mM CaCl_2_; Figure S6: Size distributions of carboxylated PAA-g-PEG self-assemblies before and after ultrafiltration; Figure S7: Time course of average particle size of carboxylated PAA-g-PEG self-assemblies in aqueous medium after ultrafiltration (polymer concentration, 5.0 mg/mL; PBS; 25 °C).
