# Supplementary material for: Calcium Ion-Induced Self-Assembly of Carboxylated Polyallylamine-graft-Poly(Ethylene Glycol) in an Aqueous Medium
Source: Polymers (Basel). 2025 Nov 25;17(23):3129. doi: 10.3390/polym17233129 (PMC12694437; doi:10.3390/polym17233129)
Supplement: Supplementary file 1 [file polymers-17-03129-s001.zip › polymers-3986760-supplementary.pdf]

Supporting Information

Calcium Ions-Induced Self-Assembly of Carboxylated Polyallylamine-graft-Poly(Ethylene Glycol) in Aqueous Medium

*Junya Emoto, Yukiya Kitayama and Atsushi Harada**

Department of Applied Chemistry, Graduate School of Engineering, Osaka Metropolitan University, 1-1, Gakuen-cho, Naka-ku, Sakai, Osaka 599-8531, Japan

Corresponding author:

Atsushi Harada

Department of Applied Chemistry, Graduate School of Engineering, Osaka Metropolitan University, 1-1, Gakuen-cho, Naka-ku, Sakai, Osaka 599-8531, Japan

Tel: +81-72-254-9328, Fax: +81-72-254-9328

Email: atsushi_harada@omu.ac.jp

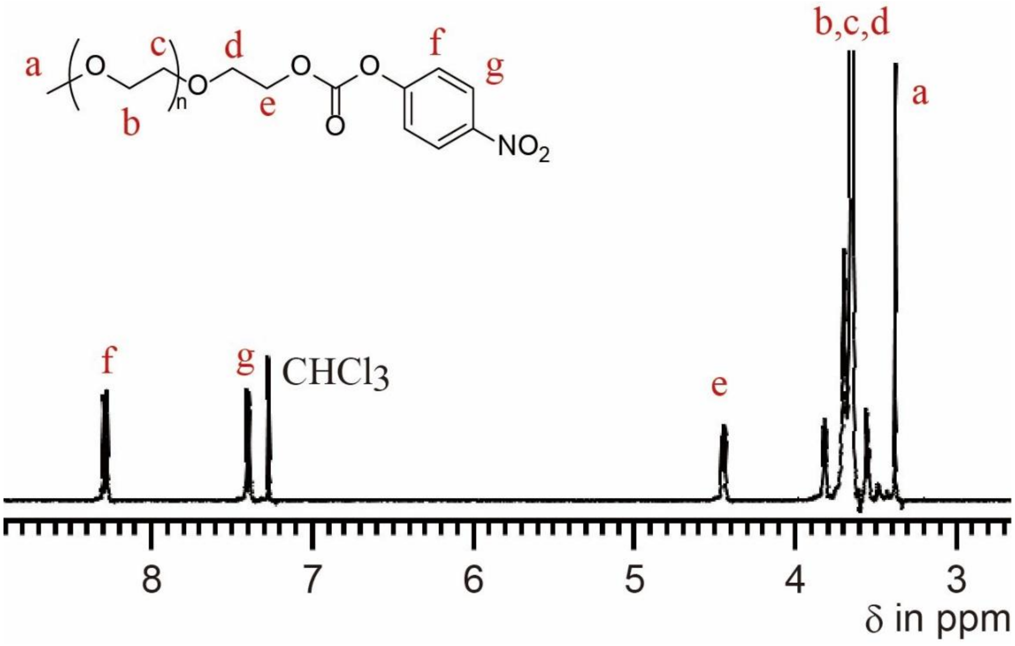

Figure S1. ^{1}H NMR spectrum of α-methyl-ω-nitrophenyl carbonate poly(ethylene glycol). 400 MHz; solvent, $CDCl_3$.

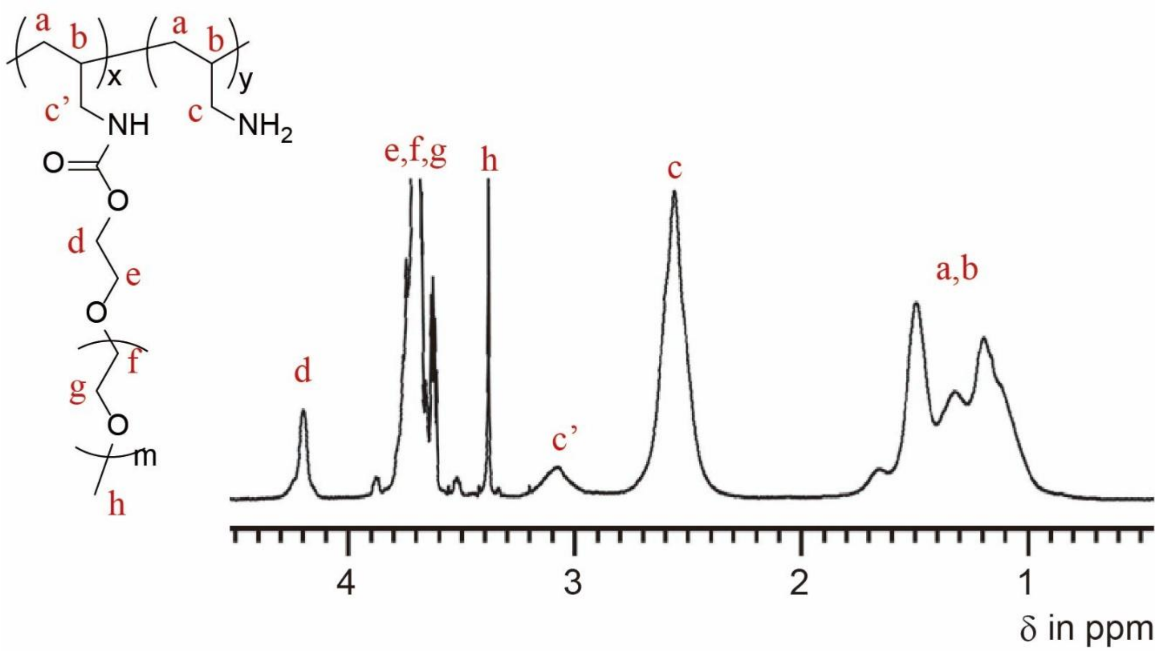

Figure S2. ^{1}H NMR spectrum of PAA-*g*-PEG. 400 MHz; solvent, D_2O with NaOD.

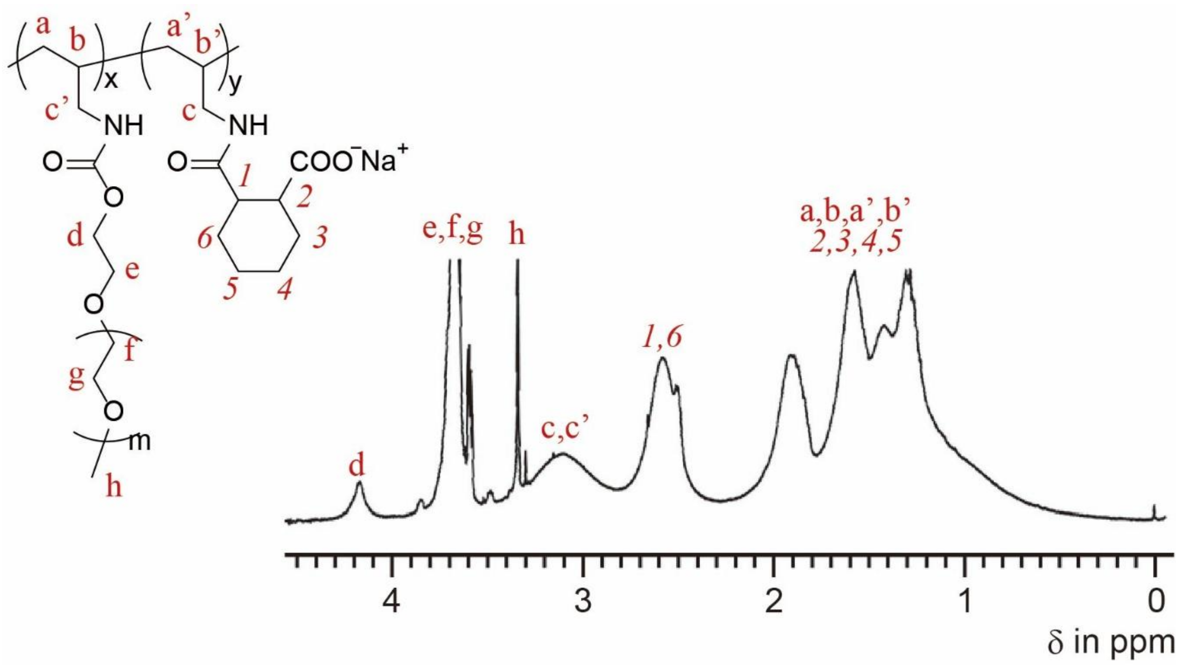

Figure S3. ^{1}H NMR spectrum of carboxylated PAA-*g*-PEG. 400 MHz; solvent, D_2O with NaOD.

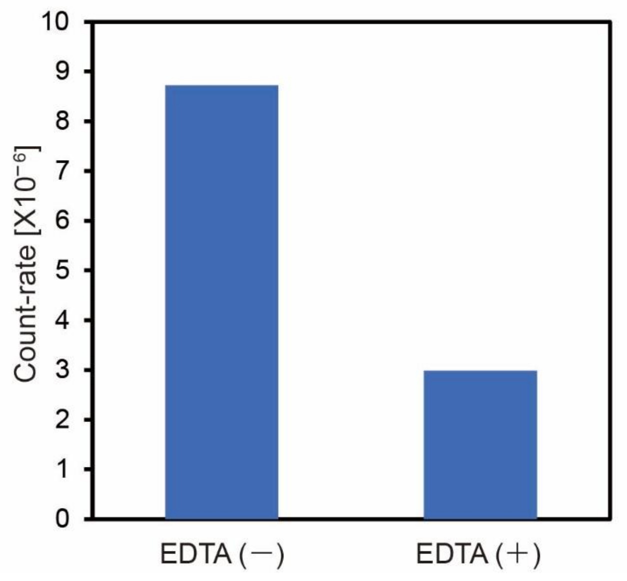

Figure S4. Count-rates of carboxylated PAA-*g*-PEG self-assemblies in $CaCl_2$ aqueous solutions with or without EDTA. $[Ca^{2+}]/[EDTA] = 1$; polymer concentration, 3.8 mg/mL; pH 7.4; 25 °C.

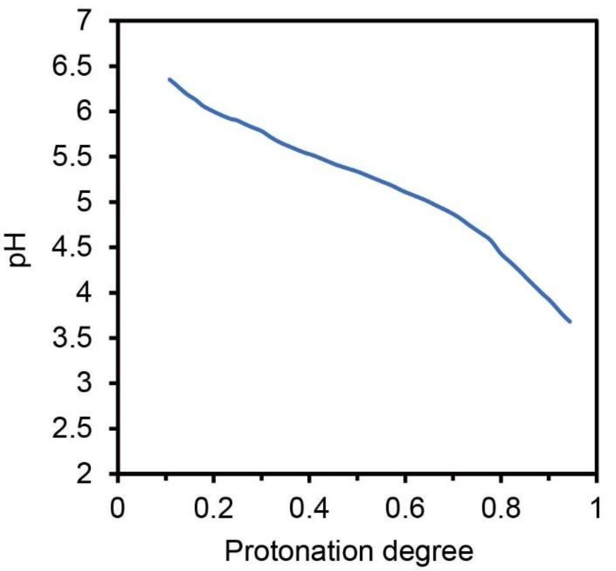

Figure S5. Acid-base titration curve of carboxylated PAA-*g*-PEG in 150 mM $CaCl_2$ at 25 °C.

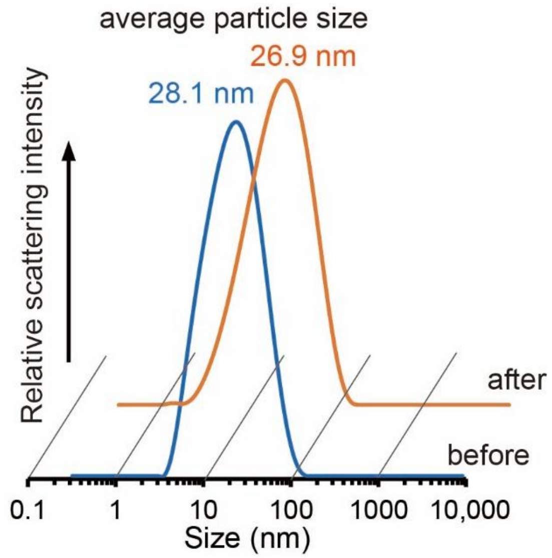

Figure S6. Size distributions of carboxylated PAA-*g*-PEG self-assemblies in aqueous medium before and after ultrafiltration (before ultrafiltration: 5.0 mg/mL, 150 mM $CaCl_2$ aq., 25 °C; after ultrafiltration: 5.0 mg/mL, PBS, 25 °C).

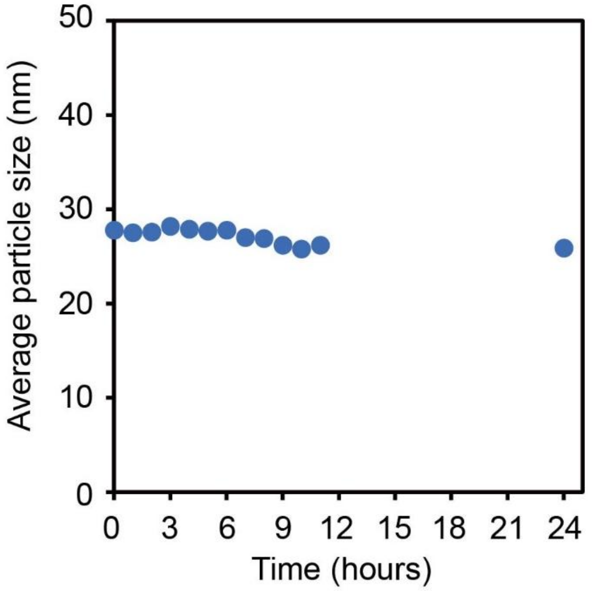

Figure S7. Time course of average particle size of carboxylated PAA-*g*-PEG self-assemblies in aqueous medium after ultrafiltration (polymer concentration, 5.0 mg/mL; PBS; 25 °C).
